# Assessment of the Corrosion of Steel Embedded in an Alkali-Activated Hybrid Concrete Exposed to Chlorides

**DOI:** 10.3390/molecules27165296

**Published:** 2022-08-19

**Authors:** William Valencia-Saavedra, Ana María Aguirre-Guerrero, Ruby Mejía de Gutiérrez

**Affiliations:** Composite Materials Group (GMC-CENM), Universidad del Valle, Cali 76001, Colombia

**Keywords:** hybrid alkali-activated cements, fly ash, granulated blast furnace slag, reinforced concretes, corrosion, chloride ions

## Abstract

Hybrid alkali-activated cements (HAACs), also known as cements with high percentages of alkali-activated supplementary materials, are alternative cements that combine the advantages of ordinary Portland cement (OPC) and alkali-activated systems. These cements are composed of a minimum of 70% precursor material and a maximum of 30% OPC mixed with an alkaline activator. This article evaluates the corrosion performance of reinforced HAAC concrete based on fly ash (FA) under exposure to chlorides (FA/OPC, 80/20). Its performance is compared with that of a binary alkali-activated cement (AAC) based on FA and granulated blast furnace slag (GBFS) (FA/GBFS, 80/20). The tests performed on the concrete matrix correspond to the compressive strength and permeability to chloride ions. Using accelerated corrosion techniques (impressed voltage) and electrochemical tests after immersion in 3.5% NaCl, the progress of the corrosive process in the reinforcing steel is evaluated. The FA/OPC exhibit a better corrosion performance than the FA/GBFS concrete. At the end of the exposure to chlorides, the FA/OPC hybrid concrete presents the best performance, with a 49% lower corrosion rate than that of the FA/GBFS. Note that according to the polarization curves, the values of the proportionality constant B in the alkaline-activated concretes differ from the values recommended for concrete based on OPC.

## 1. Introduction

Ordinary Portland cement (OPC)-based concrete has been one of the most attractive materials in the civil industry sector due to its low cost and excellent mechanical and durability properties [1]. However, today, it faces one of its greatest challenges, its environmental impact [2]. The Portland cement industry consumes extensive natural resources, such as limestone and clays, in addition to excessive energy consumption, to produce clinker. This process emits CO_2_ into the atmosphere; it is estimated that the cement industry represents 5 to 10% of global greenhouse gas emissions [2,3,4,5]. For this reason, the development of low-carbon cementitious materials that offer characteristics similar or even superior to those of traditional OPC is of great interest. These alternatives include alkali-activated materials (AAMs), geopolymers, and hybrid cementitious materials. In the process of obtaining these materials, raw materials are involved that are mostly industrial by-products, waste, and materials that may not have any commercial value. In addition, the energy requirements for their production are low; reportedly, in some cases, the use of these materials can contribute to the reduction of the carbon footprint in construction processes [6,7].

AAMs are produced through the chemical reaction of precursor material, an aluminosilicate source, and an alkaline activator. The precursor materials commonly used are fly ash (FA), metakaolin, natural pozzolans, granulated blast furnace slag (GBFS), and construction and demolition waste, among others [8,9,10]. The products of this reaction include sodium aluminosilicate hydrate (NASH)- and/or calcium silicate hydrate (CASH)-type gels, and the existence or coexistence of these gels depends on the raw materials, specifically the presence or absence of calcium in its composition [11,12,13]. In the production of some AAMs, thermal curing is necessary, and in general, temperatures between 40 and 90 °C are used; however, this technologically limits the use of these materials in the production of concretes on a large scale. It has been shown in these materials, secondary sources of calcium (CaO) and/or reactive Al_2_O_3_ can be incorporated as an alternative to replace thermal curing [14]. From this, binary alkali-activated cements (AACs) have arisen, where the content of the primary precursor material is greater than or equal to 70% and that of the by-products with calcium content (secondary precursor) is less than or equal to 30%. For the latter, the commonly used by-product is GBFS. These materials have been characterized by being free of clinker. However, in recent years, a new type of binder, known as hybrid alkali-activated cement (HAAC), has been proposed by researchers [15], and is based on the incorporation of small amounts of OPC (less than 30%) to integrate the advantages offered by OPC with those of AAMs. Due to the inclusion of OPC and because they are materials with a potentially low carbon footprint, these hybrid cementitious materials have gained the attention of the scientific community and the Portland cement industry [16,17].

As mentioned above, FA is one of the most commonly used precursor materials for the production of AAMs. Regarding the use of FA in AAMs, certain specific characteristics have been recommended (high content of glass phase, 80–90% of particles smaller than 45 µm, Fe_2_O_3_ content not exceeding 10%, reactive silica content between 40% and 50%, and less than 5% loss on ignition (LOI)) [18,19]. However, in some thermal processes, FA waste is of low quality, in particular containing high unburned contents (LOI greater than 10%). Although the standards do not allow its use as a supplementary material in mixtures with OPC [20], previous studies have demonstrated the viability of using these low-quality FA precursors in the production of AACs, obtaining promising results in terms of mechanical performance and durability in the presence of sulfates, elevated temperatures, and carbonation [19].

One of the main durability problems faced by the components of civil infrastructure that has a great influence on their useful life is the corrosion of steel in reinforced concrete structures. To maintain the structures in a safe and functional state, high-cost repairs and maintenance are necessary. OPC-based concretes provide the necessary pH conditions to promote the passivation of the reinforcing steel; however, when they are exposed to chloride ions, the ions can diffuse through the cementitious matrix, and upon reaching a critical concentration, they can degrade the passive layer and initiate corrosive processes in the reinforcing steel that can result in a rapid deterioration of the structure [21,22,23]. In AACs, reinforcing steel is passivated; however, the formation mechanisms and pH conditions are controlled by mechanisms different from those of OPC-based concretes [24]. Several studies have reported that AACs have a greater resistance to chloride ion penetration and better reinforcing steel corrosion performance [25]. Some authors state that the corrosion performance of reinforcing steel depends on different factors, such as the composition and nature of the activator solution, the curing temperature of the mortar or concrete, and the conditions to which it is exposed [26,27].

Monticelli et al. [27] studied the behaviour of steels in alkali-activated mortars based on FA type F, and exposed them to wetting-drying cycles in a 0.1 M NaCl solution, finding that activated mortars provided less corrosion protection than mortars manufactured with conventional cement. Babaee and Castel [28] compared the behaviour of AACs based on FA and concretes manufactured with cements with a high content of GBFS and reported similar behaviour. OPC-based systems do not necessarily apply to alkali-activated systems. Gunasekara et al. [29] prepared a series of samples of geopolymeric concretes that contained different percentages of chlorides (0–5%) using three types of FA. The corrosion potential (E_corr_), polarization resistance (Rp) and corrosion current (I_corr_) for the steel embedded in the geopolymer concrete during the first three months showed that the passivation of the reinforcing steel was slightly lower than that of concrete made with OPC. Other studies have shown opposite results, stating that alkali-activated mortars or concretes based on FA have a protection similar to or better than that of reinforcing steels embedded in materials manufactured with OPC, achieving similar I_corr_ and E_corr_ [27,30,31]. On the other hand, for binary alkali-activated systems based on FA, Babaee and Castel [28,32] evaluated the effect of the GBFS content (25 to 75%) in mortars and concluded that by increasing this percentage, the diffusion coefficient of chloride ions is reduced, and they highlight that mixtures with more than 50% GBFS perform better than a 100% OPC-based mortar. In the same sense, Tennakoon et al. [33] stated that in the presence of chloride ions, the corrosion resistance of the reinforcing steel of concrete based on FA-GBFS (50%/50%) was higher, coinciding with the results of Prusty and Pradhan [34], who reported lower current density values for a concrete based on 100% FA. Reportedly, the coexistence of NASH and CASH gels in these systems can contribute to reducing the permeability of the material because CASH gel has an intrinsically finer pore network than the aluminosilicate network [32].

Studies of these hybrid materials have mostly been carried out on pastes and mortars and focus on the mechanisms of hydration and the mechanical performance [35,36,37,38]. Regarding the corrosion of embedded steel in HAAC based on FA, the studies are limited, and the results are controversial [17]. In a previous study, Valencia-Saavedra et al., [19] evaluated the carbonation-induced corrosion performance of a hybrid concrete based on FA/OPC and reported current densities (I_corr_) lower than those of OPC concrete after 20 months of exposure to accelerated carbonation (1% CO_2_, 65% RH, 25 °C). Note that there are no reports on the corrosion performance of these hybrid concretes (FA/OPC, 80/20) in the presence of chlorides.

This article evaluates the corrosion behaviour of the reinforcing steel of an HAAC based on FA (FA/OPC, 80/20) in the presence of chlorides. As a reference, binary AAC (FA/GBFS, 80/20). To evaluate the corrosive process, two exposure methodologies were used: the accelerated technique known as impressed voltage and electrochemical tests (half-cell potential, linear polarization resistance (LPR), and Tafel slopes) after immersion in a 3.5% NaCl aqueous solution.

## 2. Materials and Methods

### 2.1. Materials

To produce alkaline-activation systems, FA, GBFS and OPC for general use were used. The chemical compositions of the FA, GBFS, and OPC were determined with the X-ray fluorescence (XRF) technique using a Phillips MagiX-Pro PW-2440 spectrometer, and the results obtained are shown in Table 1. The FA presents a high LOI (20.67%) that exceeds the 6% established by ASTM C618. The average particle size of the materials was determined by laser granulometry in a Mastersizer-2000 from Malvern Instruments, obtaining values of 22.1, 17.8 and 21.5 µm for the FA, GBFS and OPC, respectively.

**Figure 1:**
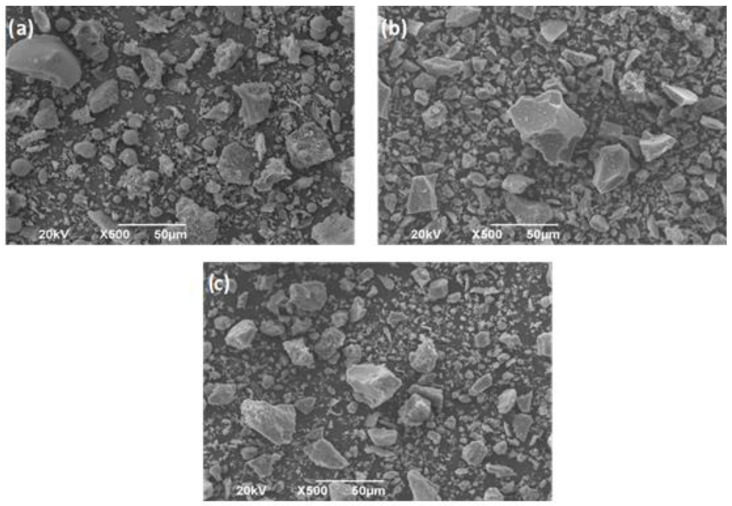
SEM micrograph of raw materials (**a**) FA, (**b**) GBFS y (**c**) OPC.

The micrographs obtained by scanning electron microscopy (SEM) for each of the materials are presented in Figure 1. This test was performed with a JEOL JSM-6490LV microscope at an acceleration voltage of 20 kV. Spherical and spheroidal particles, and irregular particles that correspond to unburned carbon can be observed in FA (Figure 1a), coinciding with reports from other studies [39,40,41,42]. The GBFS micrograph (Figure 1b) presents particles of angular shapes and variable size, which coincides with the reports of [43,44,45]. Figure 1c shows that the OPC particles are irregular and have very variable sizes, with shapes very similar to those observed for GBFS.

As an activator solution, a mixture of commercial sodium silicate (Na_2_SiO_3_·nH_2_O with chemical composition of 32.34% SiO_2_, 11.81% Na_2_O and 55.85% H_2_O) and industrial grade sodium hydroxide (NaOH, 96.7% purity) was used to obtain the appropriate molar ratios for each concrete produced.

### 2.2. Preparation of Concretes

To prepare the concretes, river sand with a fineness modulus of 3.1 and crushed gravel with a maximum particle size of 12.7 mm were used according to the ASTM specifications [46]. Two types of alkali-activated concrete (AAC) were manufactured (FA/GBFS and FA/OPC), with proportions of 80/20 (Table 2). The molar ratios for the FA/GBFS were SiO_2_/Al_2_O_3_ = 3.85 and Na_2_O/SiO_2_ = 0.25, and for the FA/OPC they were SiO_2_/Al_2_O_3_ = 4.5 and Na_2_O/SiO_2_ = 0.40. The L/S ratio was 0.48 by weight. Note that L represents the water content present in the mixture in addition to that supplied by the activator and that S includes the solid phase represented by the precursors and the anhydrous activator. The AAC samples were cured for 28 days at room temperature (26 °C) and relative humidity (RH) above 90%.

### 2.3. Experimental Tests

#### 2.3.1. Compressive Strength and Resistance to Water Penetration

The compressive strength tests were carried out according to the ASTM C39 standard using an ELE International testing machine with a capacity of 1500 kN at a speed of 1 mm/min until breakage. The resistance to water penetration (m) was evaluated following the standard procedure described in EMPA SIA 162/1 [47] and ASTM C1585 [48]. The concrete samples, once removed from the curing chamber, were dried at a temperature of 60 °C until their weight was constant, and subsequently, a coating of polymeric film (*Acronal acrylic primer*) was applied on the lateral face of the specimen to make this face impermeable to direct the entry of water. The tests were performed on cylindrical specimens (76.2 mm diameter and 152.4 mm height) at curing ages of 28 and 360 days. The values reported of compressive strength and resistance to water penetration correspond to the average of three specimens.

#### 2.3.2. Resistance to Chloride ion Penetration

Cylindrical specimens 76.2 mm in diameter and 50 mm thick were used to evaluate chloride permeability. The test was carried out based on the ASTM C1202 standard [49]. The values reported correspond to the average of three specimens and the test was carried out at curing ages of 28 and 360 days.

#### 2.3.3. Corrosion Evaluation of Steel Embedded in Concrete

**Figure 2:**
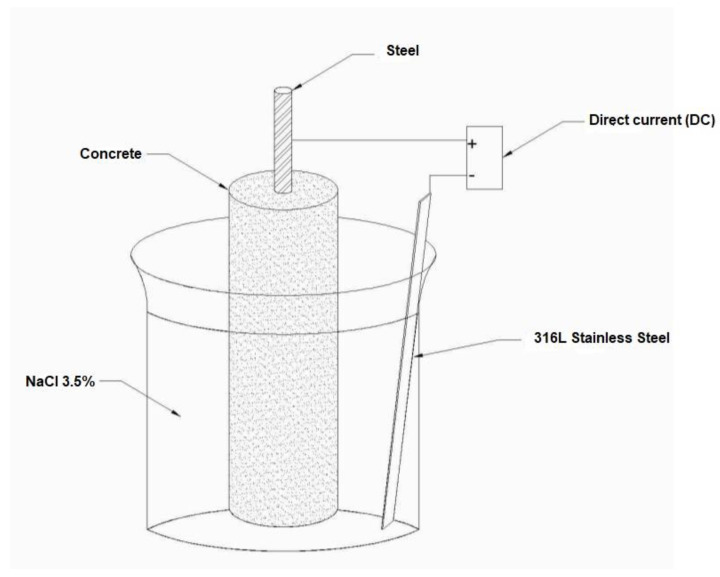
Impressed Voltage test setup.

For the evaluation of the electrochemical behavior of the reinforcing steel embedded in the studied concretes, cylindrical samples with a diameter of 76.2 mm and a height of 152.4 mm were made. The structural steel bar (AISI 1020) was placed in the central part of the specimen. This reinforcement was covered with an anticorrosive epoxy film so that the exposure area was 100 mm^2^. Several methods were used to determine the susceptibility to corrosion of structural steels in reinforced concrete:

Impressed voltage—The procedure was carried out based on the NT Build 356 standard [50], using a 3.5% NaCl solution. This accelerated method by applying an electric field determines the specimens’ failure time when exposed to chlorides. For this, a direct current (DC) is applied through the samples, at a constant potential of 5 volts. In this case, the steel rod is the positive electrode (anode) and the steel sheet (316 L stainless steel, dimensions 75 mm wide, 150 mm high and 0.90 mm thick), located to one side the specimen, is the negative electrode (cathode), as shown in Figure 2. The record of chloride diffusion is performed by measuring the current that passes through the sample during the exposure time. Based on the curve of current intensity versus time throughout the immersion process, the time required for the appearance of the first crack in the specimens was determined. To carry out the assembly, monitoring and data acquisition, an Agilent U2741A Chassis: U2781A Digital Modular Multimeter and an Agilent E3645A DC Voltage Source range from 0 to 35 V/2.2 A were used.

Immersion test—In this case, the reinforced concretes after curing for 28 days were exposed to two environments, immersion in drinking water and immersion in 3.5% NaCl aqueous solution, and the following tests were carried out to evaluate the corrosive process:

- Polarization curves (Tafel). At exposure times of 0, 180 and 360 days of exposure, the polarization curves were carried out to evaluate the electrochemical process and to determine by means of the Tafel slopes, the constant of proportionality B, according to Equation (1).

(1) $$B=\frac{\beta_{a}\beta_{c}}{2.303\left(\beta_{a}+\beta_{c}\right)}$$

where β_a_ and β_c_ correspond to the anodic and cathodic Tafel slopes, respectively.

A three-electrode electrochemical cell was used, where the working electrode corresponds to reinforcing steel, an Ag/AgCl reference electrode and a stainless-steel counter electrode located around the concrete. Overpotentials of −250 to +250 mV were applied. A Potentiostat/Galvanostat brand Autolab Instrument PGSTAT128N was used in the test.

- Linear Polarization Resistance (LPR). Linear polarization resistance was determined at the end of each month of exposure, and the test was carried out for a total period of 12 months. The LPR test was performed following the ASTM G59 standard [51]. The three-electrode electrochemical cell was used, as mentioned in the Tafel test, applying overpotentials from −30 to +30 mV. The I_corr_ corrosion rate was calculated using the Stern-Geary equation Equation (2):

(2) $$I_{\mathrm{corr}}=\frac{B}{R_{p}}$$

where B corresponds to the constant of proportionality calculated from the polarization curves for alkaline activated concretes, and R_p_ corresponds to the linear polarization resistance. For the specific OPC, the recommended value B = 26 mV was used [52].

## 3. Results and Discussion

### 3.1. Compressive Strength and Resistance to Water Penetration

**Figure 3:**
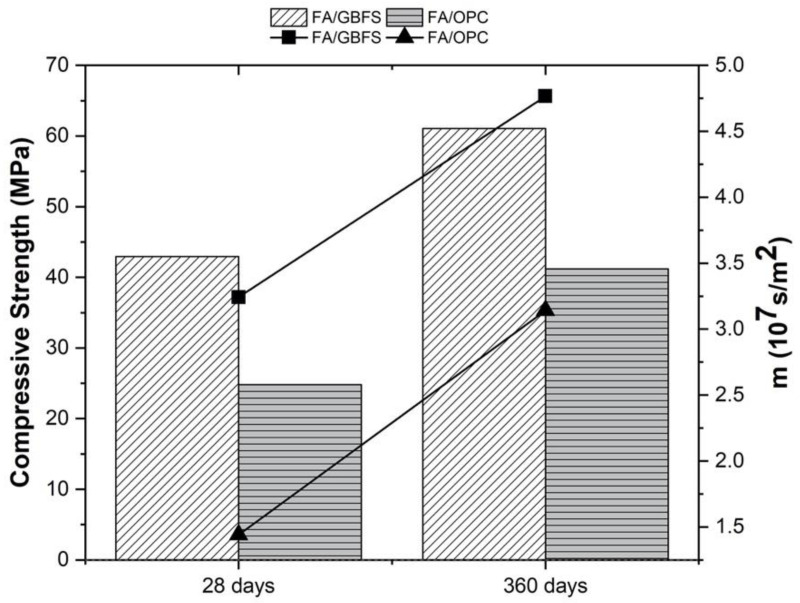
Compressive strength and Resistance to water penetration before being exposed to chlorides.

The compressive strength of the concretes FA/GBFS and FA/OPC at ages of 28 and 360 days of curing, as well as the resistance to water penetration (m), are included in Figure 3. The FA/GBFS, at a curing age of 28 days, exhibits 73% higher compressive strength and 125% higher resistance to water penetration than the FA/OPC concrete. The FA/OPC has a compressive strength of 25 MPa and a resistance to water penetration of 1.44 × 10^7^ s/m^2^. At 360 days of curing, the best behaviour is exhibited by the FA/GBFS concrete, which reaches a compressive strength of 62 MPa and a resistance to water penetration of 4.77 × 10^7^ s/m^2^. On the other hand, the FA/OPC concrete exhibit compressive strengths of 41 MPa at 360 days of curing and a resistance to water penetration of 3.14 × 10^7^ s/m^2^, surpassing by 100% the results obtained at the 28 days of curing.

**Figure 4:**
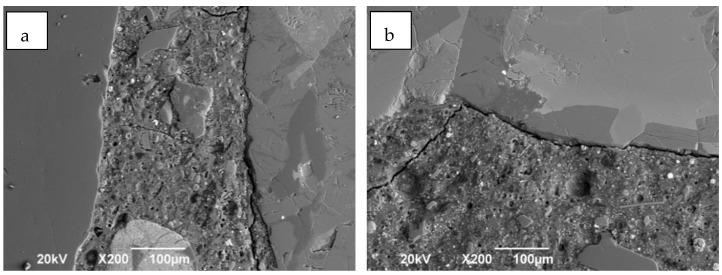
Observation matrix-aggregate interface. (**a**) FA/GBFS y (**b**) FA/OPC.

Figure 4 shows the photomicrographs of the AAC at 28 days of curing. In the FA/GBFS concretes (Figure 4a) a dense matrix with some microcracks is observed; It is noteworthy that the interfacial transition zone between the aggregate and the paste presents a good union, this good interaction coincides with the good mechanical behavior reported by the samples, as well as the greater resistance to water penetration. On the contrary, the FA/OPC concretes (Figure 4b) present a matrix with microcracks and the presence of pores. In addition, a separation or crack is observed around the aggregate in the interfacial transition zone, to which the lower compressive strength and lower resistance to water penetration can be attributed.

### 3.2. Chloride Permeability

**Figure 5:**
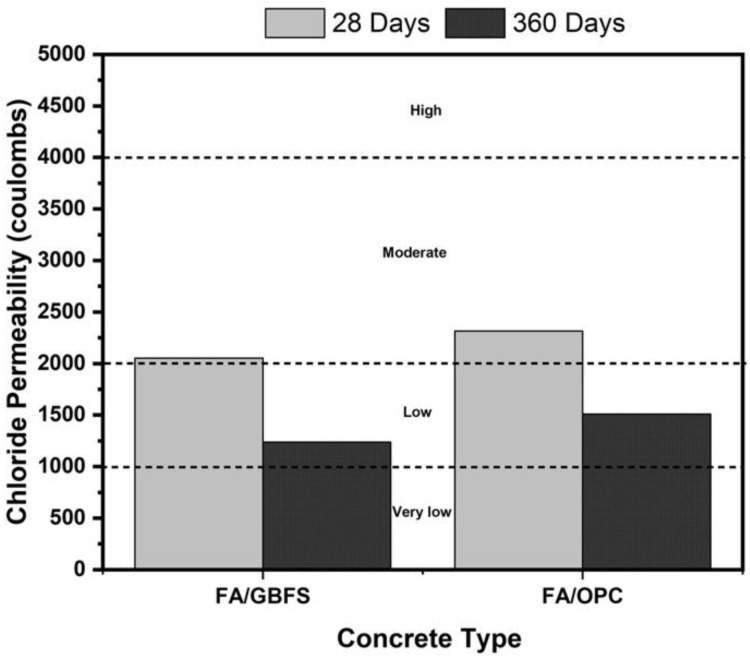
Rapid chloride permeability of FA/GBFS, FA/OPC concretes at 28 and 360 curing days.

In Figure 5, the values of the total transferred load expressed in coulombs for the concretes under study are presented. In general, a decrease in chloride permeability with curing time is observed for all mixtures. The FA/OPC concretes show the lowest resistance to chloride ion penetration of the concretes. At the age of 28 days, all samples are in the moderate permeability range with respect to the values specified in the standard ASTM C1202 [49], with charge values of 2053 C and 2315 C for the FA/GBFS and FA/OPC concretes, respectively. However, at the age of 360 days of curing, the AACs are in the low permeability range. FA/GBFS concrete stands out, with a charge of 1236 C, which indicates a low permeability to chlorides. In the FA/OPC hybrid concrete, the permeability is greater than that of the FA/GBFS samples by 22% at 360 days of curing, reaching values of 1509 C.

In the interpretation of the data obtained by RCPT, it is important to take into account that this test is fundamentally a measure of the electrical conductivity of the concretes. Ionic transport depends to a large extent on the structure of the pore network in the cementitious matrix, while the electrical conductivity in the concrete is affected both by the structure of the pore network and by the chemical composition of the solutions in the pores [53,54,55]. It has been identified that pore solutions in alkaline activation systems contain high concentrations of ionic species, mainly Na^+^ and OH^−^ [56,57,58]. The RCPT test indicates the movement of all the ions in the structure, not just the chloride ions; thereby, the results of the ASTM C1202 test for AACs could be affected. Any Na^+^ ions present in the pores of the material are expected to diffuse in the direction opposite that of the Cl^−^ ions through the pore network, which can lead to a greater charge transfer during the test. Therefore, a greater ionic strength in the pore solution of the samples is expected to lead to higher charge values, which means that the values observed in the evaluated AACs could be higher than the chloride permeability in reality.

### 3.3. Impressed Voltage

To analyze the corrosion susceptibility of the FA/GBFS and FA/OPC concretes, impressed voltage tests were carried out, through which the behavior against corrosion was rapidly evaluated. During the test, the electrical potential applied to the reinforcing bars attracts the negatively charged chloride ions from the aqueous solution of 3.5% NaCl to the concrete and towards the positively charged steel bars, thus initiating the diffusion process of chloride ions in the matrix and inducing the corrosive process [22]. Due to the corrosion in the reinforcement bar, the development of cracks generates greater access to the surface of the steel for the ions, which creates a direct current path between the reinforcement and the electrodes in the solution. Any sudden increase in current flow indicates a reduction in the electrical resistance of the concrete.

**Figure 6:**
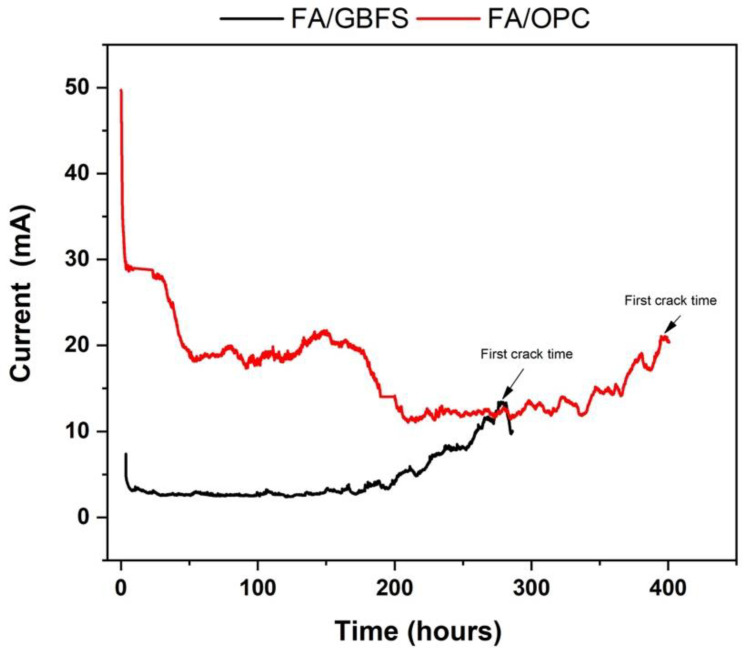
Current intensity curve versus the test time on reinforced concrete (FA/GBFS and FA/OPC).

Figure 6 shows the current intensity curve versus the test time in the FA/GBFS and FA/OPC concretes. At a general level, the current intensity for the alkaline activation concretes presents high initial values (FA/GBFS = 10 mA and FA/OPC = 50 mA) and subsequently decreases, reaching 3 mA in the FA/GBFS samples at 7 h and 11 mA in the FA/OPC samples at 209 h. These initial high current values can be attributed to the high ionic concentrations of Na^+^ and OH^−^ present in the pore solution of the alkaline activation concretes [59]; coincidently, the specimens that contain the greatest amount of Na+ (FA/OPC) present a greater initial current and take longer to stabilize. This last effect could be attributed to a possible binding of the chloride ions in the gels (CASH, NASH) as a result of alkaline activation, which impedes the passage of charge flow [60]. The subsequent increase in current intensity is directly related to the progress of the corrosion of the steel and therefore to the appearance of cracks as a consequence of the tensile stresses in the hardened matrix due to the presence of the corrosion products.

The FA/OPC concretes present a higher current intensity than the FA/GBFS at the appearance of the first crack, reaching values of 20 mA, as is presented in Table 3. In the case of the FA/GBFS, the maximum current value reached is 12 mA. The current intensities of the FA/GBFS concrete are 40% lower than that of the FA/OPC concrete. In this study, the FA/OPC-based specimens take ~398 h to crack, while in the case of the FA/GBFS specimens, the times is ~281. The good performance of the alkaline activation concretes is an indication of the lower permeability to chloride ions that they present, which is reflected in the lower susceptibility to corrosion of the reinforcing bars and leads to a significant improvement in the strength. This behaviour in alkaline activation concretes was also observed by Reddy et al. [61]. However, in this test, the hybrid concrete (FA/OPC) presented better performance, extending the appearance of the first crack for a longer time.

**Figure 7:**
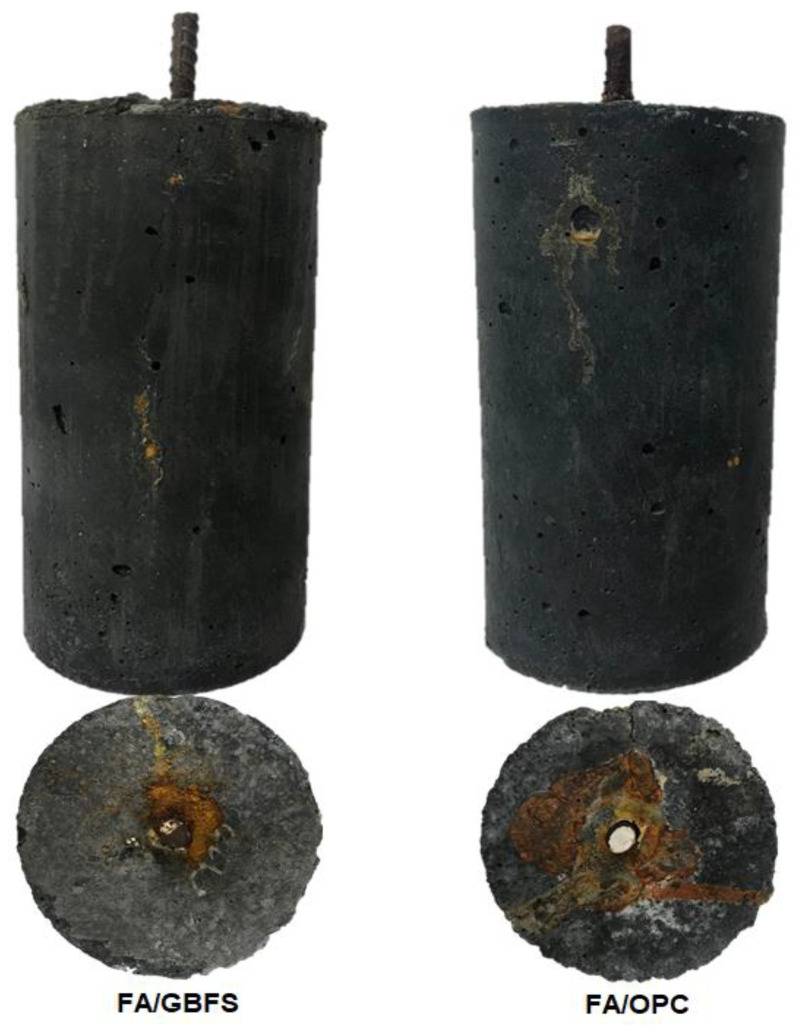
Photographs of the concretes after the impressed voltage test.

Figure 7 shows the photographs corresponding to the reinforced concretes after the impressed voltage test. The first visual evidence of corrosion corresponds to the appearance of brown spots on the surface of the specimen. Cracking is observed shortly thereafter. In the analysis of the sizes of the cracks for the different concretes studied (Table 3), it is observed that in the FA/OPC concrete, the cracks are sized 0.25 mm, which is the highest value reported for the mixtures analysed. The FA/GBFS concretes have a crack size of 0.18 mm. According to ACI 318-11 (ACI 2011), a concrete material is in a state of failure when the crack widths on its surface reach 0.3 mm. Within this context, it can be said that the alkaline activation samples are not in a state of failure, given the where the width of the failure does not exceed 0.25 mm.

**Figure 8:**
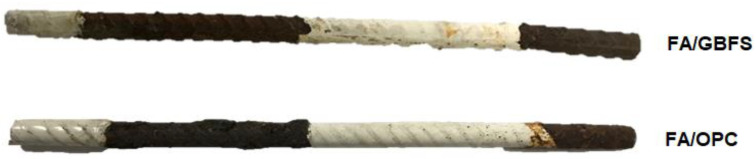
Rebar bars recovered after accelerated corrosion tests.

It is well known that the most reliable method for determining the degree of corrosion in reinforcing steel is a mass loss measurement. Therefore, to determine the mass loss of the corroded reinforcing steel, the concrete samples were transversely broken to extract the reinforcing bar. Figure 8 shows the state of the reinforcement steels after extraction from the concretes. According to ASTM G102 [62], it was necessary to remove all corrosion products from the reinforcing steels before weighing them. Therefore, a 10% sulfuric acid solution was used to perform a pickling of the samples, and a metal brush was used to remove any remaining corrosion product. The bar embedded in the FA/OPC concrete shows higher damage than the bar embedded in FA/GBFS concrete.

Table 4 shows that the percentage loss of mass of the steels embedded in the FA/OPC concretes after the accelerated corrosion test is 5.7%, while the FA/GBFS present mass losses of 2.7%. This is directly related to the larger size of the longitudinal cracks that are observed in the FA/OPC concretes (Figure 7), which in turn contribute to accelerating the corrosion rate by allowing greater entry of chlorides into the concrete.

### 3.4. Immersion in Drinking Water and NaCl 3.5%

#### 3.4.1. Polarization Curves

**Figure 9:**
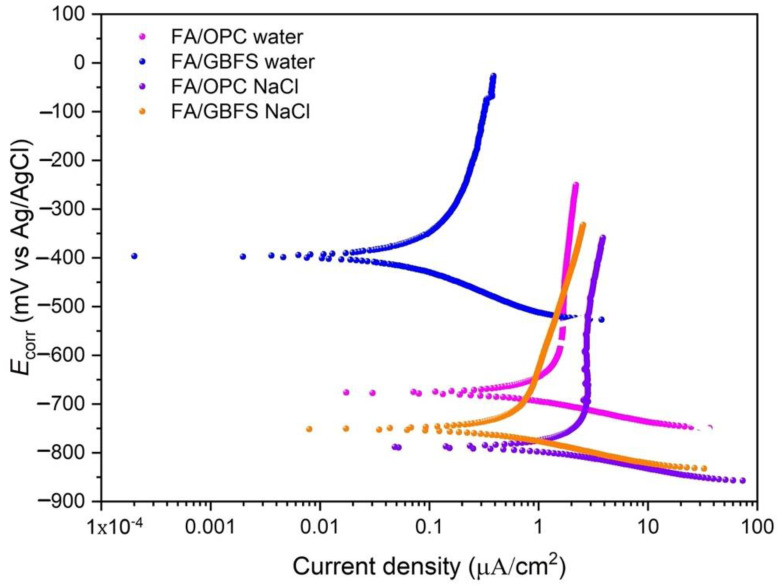
Polarization curves of AACs at 360 days of exposure in water and NaCl environments.

Figure 9 shows the polarization curves of the concretes at the end of the exposure (360 days). The corrosion potential E_corr_, the current density I_corr_ and the proportionality constant B were obtained by the extrapolation method from the polarization curves obtained in the Tafel test. These results are presented in Table 5, showing that the alkaline activation concretes, at the beginning of the exposure (0 days), have negative E_corr_ values.

This behavior has been reported by different researchers and is more marked in concretes containing GBFS due to the sulphur content of the slag, which can decrease the potential values in the redox reactions [63]. Although, for the FA/GBFS concretes high current density values are observed at the beginning, the values decrease as it is exposed in the two environments, which can be attributed to the high number of free ions in the pore solution (Na^+^, OH^−^), this can cause a higher conductivity and therefore more negative potentials and higher corrosion densities. The electrical conductivity generated by these ions is related to moisture since it increases their mobility. Bastidas et al. [64] report that the values of E_corr_ of mortars activated with FA in humid environments are more negative than those in dry environments. It is important to mention that the RH at which these tests were performed is on the order of 70% (corresponding to the atmospheric humidity of the region). Another factor mentioned in the literature that can affect the values of corrosion potentials and rates in alkaline activation systems is the pH of the pore solution; however, more studies are necessary to corroborate this effect [28]. It was observed that at 180 and 360 days of exposure, the values of I_corr_ decrease, which indicates that the ionic conductivity is reduced. This behavior coincides with that observed in the impressed voltage test (Figure 6), with high current values at the beginning of the test; however, after a certain time, these current values decrease and stabilize. In a previous study, the same behavior was observed in alkaline activation systems based on natural pozzolans [65].

The proportionality constant B was calculated from the slopes of the anodic curve (β_a_) and the cathodic curve (β_c_) Equation (1). The technical committee RILEM TC154 [52] suggests using a B value of 26 mV to perform I_corr_ calculations by means of the LPR technique for OPC concretes. According to Table 5, the alkaline activation concretes (FA/GBFS and FA/OPC) have B values different from those identified for OPC concrete, attributable to their different composition and microstructure. Babaee and Castel [28] reported values for the constant B for alkaline activated concretes with low calcium fly ash of 13 < B < 20 for the passive state and 45 < B < 58 for the active state. Aguirre-Guerrero et al. [65] reported B values of 17 mV for natural pozzolan/GBFS concretes exposed to chlorides. These values do not agree with what is reported in the present study. The chemical composition, type of precursors, the nature and proportion of activators, ions present in the pore solution, and microstructure all lead to differences in the electrochemical behaviour of the reinforcing steel embedded in alkaline activation concretes compared to OPC-based concretes. To determine I_corr_ by means of the LPR technique, the average of the B values obtained at exposure ages of 180 and 360 days of the FA/OPC and FA/GBFS concretes was calculated for the two environments evaluated. A B value of 14.6 mV ± 1.6 is obtained.

As expected, the drinking water environment is less aggressive than the environment rich in chloride ions. In general, an increase in the corrosion current values is observed as a consequence of the greater diffusion of chloride ions through the capillary pores of the materials towards the reinforcing steel in the case of the FA/OPC concrete. The concrete that presented the best performance is FA/GBFS, with the lowest current densities in the two exposure environments. The performance of the reinforced concretes exposed to chlorides coincides with that observed in the permeability to chlorides, where the FA/GBFS concrete presents the lowest permeability. This performance is according to the more densified matrix, smaller microcracks and better interfacial transition zone between the aggregate and the paste observed in FA/GBFS.

#### 3.4.2. Linear Polarization Resistance (LPR)

**Figure 10:**
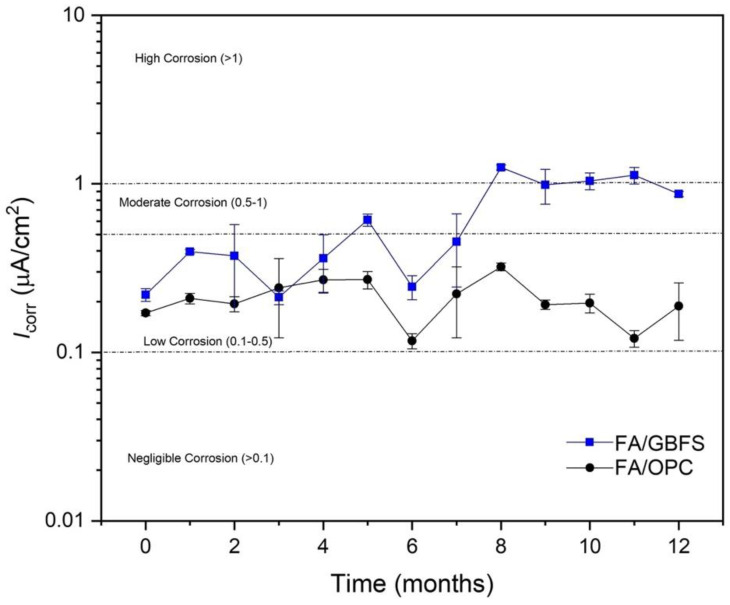
Current density (I_corr_) of specimens exposed to drinking water.

As mentioned above, B values of 14.6 mV for FA/OPC and FA/GBFS were used to determine the corrosion rate. Figure 10 shows the trend of the corrosion rate over time of the concretes exposed to drinking water. Before exposure (0 days), the FA/OPC and FA/GBFS concretes show corrosion rates that indicate depassivation of the reinforcing steel, coinciding with that observed in the polarization curves. Here, it can be observed that FA/OPC presents lower corrosion rates during the entire exposure, and it remains in the low corrosion range. However, the FA/GBFS concrete after one month shows a higher current density than the FA/OPC concrete.

**Figure 11:**
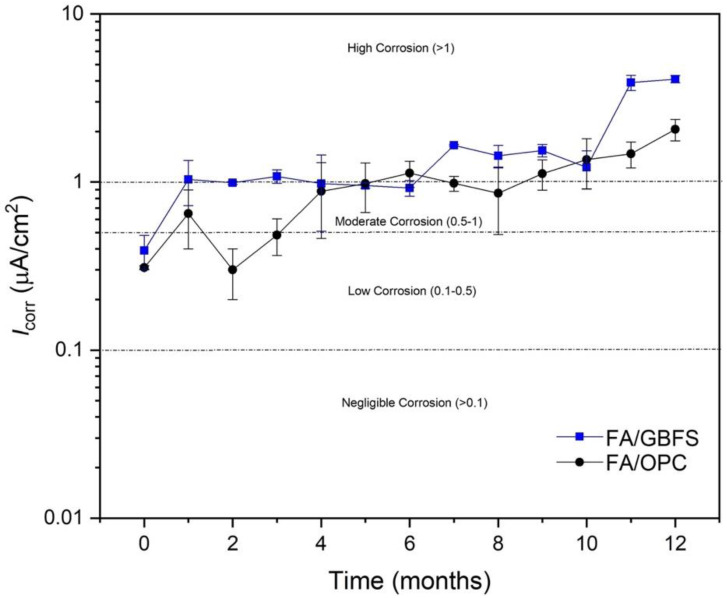
Current density (I_corr_) of specimens exposed to 3.5% NaCl.

Figure 11 shows the current density of the specimens exposed to chlorides. The values of I_corr_ increase significantly with respect to those observed in Figure 10, which corroborates that this environment is a much more aggressive environment for reinforced concretes. The concretes after the first month of exposure exhibit corrosion rates that indicate depassivation of the reinforcing steel. Up to three months, the FA/GBFS concrete presents higher corrosion rates than the FA/OPC concrete. Between months 4 and 6 alkali-activated concretes have a similar tendency initially, located in the zone moderate corrosion. At the end of the exposure (12 months), the FA/OPC concrete has a 49% lower current density than the FA/GBFS concrete.

Authors such as Tennakoon et al. [33] studied the corrosion performance of alkali-activated concretes based on FA/GBFS in proportions 50/50 under exposure to chlorides and reported lower corrosion rates compared to OPC concrete, which is in agreement with the FA/GBFS concrete exhibiting the best corrosion performance in the present study. In the literature, to date, there have been no reports of the corrosion performance of alkaline activated hybrid concretes (HAACs) such as FA/OPC under exposure to chlorides. In the present study, it can be observed that this concrete presents a better performance in the impressed voltage and LPR tests than the FA/GBFS concrete. The two alternative concretes exhibit lower corrosion rates, suggesting an extension of the useful life of the material compared to that of a conventional OPC concrete found in the literature. The microstructure of activated concretes with high Ca content form NASH- and CASH-type gels, which have been reported to be able to physically bind chloride ions in their structure, thus decreasing the probability of corrosion of the reinforcing steel [60]. Additionally, the FA/OPC hybrid concrete, with OPC in its composition, can generate calcium silica hydrate (CSH) gel products from its hydration processes. It is possible that the coexistence of these gels in this type of concrete can increase the chloride ion binding capacity and therefore exhibit better behaviour than FA/GBFS binary concrete. It should be noted that the chloride ion binding capacity depends on the composition of the precursor and activator, the concentration of the chloride solution which they are exposed, and especially the types of gels that are formed in these materials, N-A-S-H and/or C-A-S-H type.

**Figure 12:**
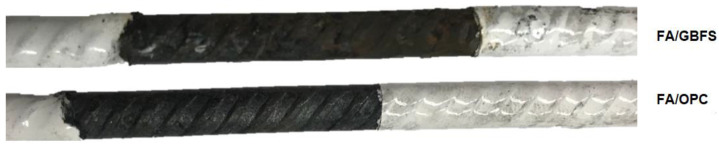
Photographs of reinforcing steels extracted from FA/GBFS and FA/OPC concretes, at the end of exposure to a 3.5% NaCl solution.

Figure 12 shows the state of the steels after exposure to 3.5% NaCl. Here, it can be seen that the steel embedded in the FA/GBFS concrete presents a small pitting and corrosion products on its surface. In contrast, the steels embedded in the FA/OPC do not show any effects. According to the values of I_corr_ reported in Figure 10, the steels embedded in the FA/GBFS and FA/OPC concretes should show greater deterioration; however, note that the ranges of values reported in this figure to evaluate the severity of corrosion correspond to those recommended for OPC concretes according to the technical committee RILEM TC154, and these do not necessarily apply to alkaline activation concretes. Authors such as Babaee and Castel [28] suggest a recalibration of the corrosion severity criteria for this type of alternative concrete. Agreeing with this, Tennakoon et al. [33] report that the values of the electrochemical measurements in alkaline activation systems do not corroborate the real corrosion activity of the system.

## 4. Conclusions

From the results obtained, the following conclusions can be drawn:

The binary FA/GBFS concrete presents higher compressive strength and resistance to water penetration at 28 days and 360 days than the FA/OPC concrete; additionally report higher resistance to chloride ion penetration. However, it is observed that at 360 days of curing, the FA/OPC concretes reach a resistance of 41 MPa, and the resistance to water penetration increases by 118% with respect to the samples cured at 28 days.

Although the FA/OPC concrete presents higher chloride permeability than the FA/GBFS concrete, in the impressed voltage test presents the best performance. In general, at the end of the test, the mass loss of the steel in FA/OPC and FA/GBFS was 5.74% and 2.75%, respectively.

The constant “B” used for the calculation of the current density I_corr_ in the linear polarization technique diverges from the typical values used in OPC concretes. These values must be calculated for concretes with different chemical compositions, such as alkaline activation systems. In this study, the values of the constant B for the FA/GBFS and FA/OPC concretes is 14.6 mV.

The FA/OPC concrete offer better resistance to corrosion of the reinforcing steel than FA/GBFS under exposure to chlorides (immersion in NaCl 3.5%). At the end of the exposure, the values of I_corr_ observed for the FA/OPC concretes are 49% lower than that for FA/GBFS concrete.

The visual inspection of the steels after exposure to chlorides for a period of 12 months does not reflect the severity of the conditions indicated by the results of the electrochemical measurements for the activated concretes, suggesting the need to modify the ranges used to evaluate the severity of corrosion in this type of concrete is needed.

## Figures and Tables

**Table 1:** Chemical composition of raw materials (%wt.).

Material	SiO_2_	Al_2_O_3_	Fe_2_O_3_	CaO	MgO	Na_2_O	SO_3_	TiO_2_	LOI *
FA	28.53	19.18	8.80	6.68	2.24	7.94	2.71	1.62	20.67
GBFS	31.99	14.54	1.12	46.86	1.05	0.23	0.82	0.54	1.82
OPC	19.13	4.42	4.32	57.70	1.60	-	2.32	-	9.78

* Loss of ignition.

**Table 2:** Concrete design (kg/m^3^) and liquid/solid ratio.

Mixtures	Cement (kg)	FA (kg)	GBFS (kg)	NaOH (kg)	SS (kg)	Sand (kg)	Gravel (kg)	L/S Ratio
FA/GBFS	0	320	80	28.5	158.4	972	704	0.48
FA/OPC	80	320	0	48.4	219.7	972	704	0.48

**Table 3:** Results of Impressed voltage test on reinforced concrete (FA/GBFS, FA/OPC and OPC).

Mixture	Cracking Time (h)	Maximum Anodic Current at the Time of the First Crack (mA)	Crack Size (mm)
FA/GBFS	281	12	0.18
FA/OPC	398	20	0.25

**Table 4:** Percentage loss of mass of the steels embedded in the concretes after the accelerated.

Sample	Loss of Mass (gr)	Loss of Mass (%)
FA/GBFS	−0.92	2.75
FA/OPC	−1.92	5.74

**Table 5:** Corrosion potential (E_corr_), current density (I_corr_) and constant of proportionality (B) of the reinforced concretes.

Concrete	Exposition Environment	Exposition Time (Days)	E_corr_ (mV Vs Ag/AgCl)	I_corr_ (μA/cm^2^)	B
FA/OPC	Water	0	−179	0.052	19.2
		180	−514	0.092	13.2
		360	−674	0.794	17.1
	NaCl (3.5%)	0	−218	0.074	20.0
		180	−643	0.987	12.7
		360	−788	1.383	13.9
FA/GBFS	Water	0	−600	0.612	22.1
		180	−400	0.133	15.6
		360	−397	0.062	16.6
	NaCl (3.5%)	0	−657	0.627	24.1
		180	−609	0.182	13.6
		360	−750	0.476	14.4
